# A Case of Priapism with Risperidone

**DOI:** 10.1155/2014/241573

**Published:** 2014-10-14

**Authors:** Almari Ginory, Mathew Nguyen

**Affiliations:** Department of Psychiatry, University of Florida, Gainesville, FL 32610, USA

## Abstract

Priapism is a urologic emergency defined as a prolonged, possibly painful, penile erection. There are several known causes of priapism including psychotropic medications. One of the mechanisms by which antipsychotics are believed to induce priapism is through alpha-1 antagonism. This is case of a 50-year-old male with a history of schizophrenia with previous priapism related to trazodone, who presents with new onset priapism associated with risperidone. In this case, the treatment of priapism includes discontinuation of the offending agent and drainage of the corpus cavernosum twice along with intracavernosal phenylephrine injections. It is important to educate patients on priapism as a possible side effect of medications. It is also important to consider previous episodes of medication-induced priapism when prescribing psychotropic medications as this may increase the patient's future risk of priapism.

## 1. Introduction

Priapism is defined as greater than 4 hours of penile erection not initiated by sexual stimulation [1]. There are several causes of priapism including medications, hematologic disorders, substances, malignancies, trauma, and metabolic conditions [1, 2], Table 1. Priapism is considered a urologic emergency which, if left untreated, could lead to impotence and tissue necrosis [3]. The treatment of priapism includes conservative management (observation, ice, and rest), corporal aspiration, injection of sympathomimetic agents, and, if the above treatments fail, surgical intervention [4]. Antipsychotics are hypothesized to cause priapism through alpha-1 adrenergic antagonism [3]. Herein we present a case of priapism caused by risperidone in a patient with a previous episode of priapism caused by trazodone.

## 2. Case Report

Mr. R is a 50-year-old Caucasian male with a prior psychiatric history of schizophrenia and no known past medical history. He was admitted to the hospital for 18 hours of penile erection associated with swelling and severe throbbing pain. He denied any history of illicit substance use, hematological illnesses, or penile trauma. His only prescription medication was risperidone 3 mg twice daily, which was started 1 month before. He reported a previous episode of similar symptoms approximately 5 years ago associated with trazodone which was prescribed for insomnia. He delayed in presenting to the emergency room because he did not know that this could be a side effect from his medication and assumed it would resolve on its own. On evaluation, his urine drug screen was negative, as were the remainder of his labs except for a mildly elevated white blood cell count (10.8 thou/cu mm, normal range is 4.0–10.0 thou/cu mm).

Mr. R was evaluated by urology and was diagnosed with nonischemic priapism secondary to psychotropic medications. He was admitted to the hospital and underwent drainage of the corpus cavernosum twice along with intracavernosal phenylephrine injections. Psychiatry was consulted for medication recommendations. At the time of evaluation, he did not report symptoms of depression, mania, psychosis, or anxiety. Historically, his symptoms of psychosis included both auditory and visual hallucinations, which he has not experienced in over a year. He described the auditory hallucination as a single male voice that was derogatory in nature. The visual hallucinations were of deceased relatives. He had no prior inpatient psychiatric hospitalizations or treatment with other antipsychotic medications. He experienced the hallucinations over several years. The decision was made to discontinue risperidone due to side effects. Since he has no acute symptoms and had a prior episode of priapism with a different medication, he was not started on any new medications and was referred for outpatient followup. His symptoms of priapism completely resolved 24 hours after initial presentation at the hospital.

## 3. Discussion

While priapism is an uncommon side effect of antipsychotics, it is one that patients should be made aware of due to the possible complications. The proposed mechanism by which antipsychotics are hypothesized to cause priapism is through alpha-1 adrenergic antagonism [3]. Both atypical and typical antipsychotics have been associated with priapism [2]. For the typical antipsychotics, more cases have been reported with low potency agents, such as chlorpromazine, compared to the high potency agents, such as haloperidol, due to the level of alpha-1 adrenergic antagonism [2]. For the atypical antipsychotics, risperidone and ziprasidone have the highest antagonism at alpha-1, and olanzapine has the lowest [3]. However, there have been several published cases of priapism with other atypical antipsychotics with lower alpha-1 antagonism such as olanzapine, quetiapine, and clozapine [5]. Risk factors for development of priapism include recent dose changes, recent medication changes, reinitiation of medication after periods of noncompliance, concomitant substance use, and/or use of other medications which also cause priapism [2]. Priapism can occur at any point in treatment as it has not been found to be associated with either duration of treatment or dose of medication [6].

There is some evidence to suggest that patients with prior episodes of priapism are at increased risk of subsequent episodes of priapism [7]. Patients, such as this one with long histories of psychiatric symptoms and adverse reactions to more than 1 medication, present a difficult treatment conundrum for providers. In patients with a history of priapism associated with trazodone or other antipsychotics, an antipsychotic with less alpha-1 adrenergic antagonism should have been considered while still educating the patient the possibility that priapism could recur. A case report by Penaskovic et al. illustrated a case of priapism with several atypical antipsychotics (risperidone, quetiapine, and olanzapine) which was stabilized on loxapine with no further instances of priapism [7]. A thorough discussion of risk versus benefit of treatment should be had with the patient as well as family, if indicated and consented to by the patient.

This case illustrated the importance of proper patient education on side effects as well as obtaining complete historical information on side effects from medications in order to prevent recurrence. In this case, due to the severity of Mr. R's priapism and the absence of psychiatric symptoms, the decision was made to not start medications as the risks of side effects outweighed possible benefits. In other cases, a medication with less alpha-1 antagonism should be considered and used at the lowest effective dose [8]. Patients should be educated on the possible risk of priapism, including future episodes, and to present to the hospital emergently should symptoms develop.

## Figures and Tables

**Table 1:** Common causes of priapism [1, 2].

Medications	Antihypertensive, anticoagulants, antipsychotics, and antidepressants
Metabolic	Diabetes
Hematologic	Sickle cell disease, leukemia
Substances	Alcohol, cocaine
